# MMP-9 Contributes to Dendritic Spine Remodeling Following Traumatic Brain Injury

**DOI:** 10.1155/2019/3259295

**Published:** 2019-05-06

**Authors:** Barbara Pijet, Marzena Stefaniuk, Leszek Kaczmarek

**Affiliations:** Laboratory of Neurobiology, BRAINCITY, Nencki Institute of Experimental Biology of Polish Academy of Sciences, Pasteura 3, 02-093 Warsaw, Poland

## Abstract

Traumatic brain injury (TBI) occurs when a blow to the head causes brain damage. Apart from physical trauma, it causes a wide range of cognitive, behavioral, and emotional deficits including impairments in learning and memory. On neuronal level, TBI may lead to circuitry remodeling and in effect imbalance between excitatory and inhibitory neurotransmissions. Such change in brain homeostasis may often lead to brain disorders. The basic units of neuronal connectivity are dendritic spines that are tiny protrusions forming synapses between two cells in a network. Spines are dynamic structures that undergo morphological transformation throughout life. Their shape is strictly related to an on/off state of synapse and the strength of synaptic transmission. Matrix metalloproteinase-9 (MMP-9) is an extrasynaptically operating enzyme that plays a role in spine remodeling and has been reported to be activated upon TBI. The aim of the present study was to evaluate the influence of MMP-9 on dendritic spine density and morphology following controlled cortical impact (CCI) as animal model of TBI. We examined spine density and dendritic spine shape in the cerebral cortex and the hippocampus. CCI caused a marked decrease in spine density as well as spine shrinkage in the cerebral cortex ipsilateral to the injury, when compared to sham animals and contralateral side both 1 day and 1 week after the insult. Decreased spine density was also observed in the dentate gyrus of the hippocampus; however, in contrast to the cerebral cortex, spines in the DG became more filopodia-like. In mice lacking MMP-9, no effects of TBI on spine density and morphology were observed.

## 1. Introduction

Traumatic brain injury caused by an external mechanical force evokes a variety of brain responses, including focal extrasynaptic matrix degradation, neuronal loss within hippocampus area, glia activation, synaptic remodeling, and ion channels activity changes [1, 2]. On a neurotransmission level, a massive glutamate efflux, increased level of extracellular glutamate, and hyperactivation of NMDAR receptor channels followed by their loss are observed [3]. These events are also strictly related to dendritic spine remodeling [4]. Dendritic spines are small membranous protrusions that undergo plastic morphological changes under both physiological (e.g., development or learning and memory) and pathological (e.g., neurodegeneration, psychiatric disorders) conditions [5–7]. Several recent reports have described changes in dendritic spine density and size following brain trauma [8–16].

MMP-9 is pericellularly acting endopeptidase, classified as a gelatinase due to its ability to cleave gelatin [17–21]. Through involvement in extracellular matrix remodeling, it regulates numerous cell processes and physiological functions [22–26]. Aside from physiological role, MMP-9 takes part in such central nervous system pathological events as injury, stroke, or epileptogenesis, as well as neuropsychiatric disorders such as schizophrenia or addiction [27–35]. Importantly, previous reports have also indicated that MMP-9 is a crucial dendritic spine shape modulator [36–41] and its level is altered posttrauma [28, 40, 42–45]. To what extent upon brain injury MMP-9 is involved in altering dendritic spines number and shape is yet unknown. To bridge this gap, we set out to analyze the effects of MMP-9 levels on TBI-stimulated plastic changes of the dendritic spines in the mouse brain. For this, we used controlled cortical impact (CCI) as an animal model of traumatic brain injury [46]. First, we describe influence of CCI on density and morphology of dendritic spines in the cerebral cortex and hippocampus 24 hours and 7 days of postbrain injury. Next, we assess the effects of missing MMP-9 due to the gene knockout (KO) on spine density and shape following TBI.

## 2. Materials and Methods

### 2.1. Animals

The study was performed on adult (12–14 weeks old) C57BL/6J male mice (Animal House, Center for Experimental Medicine, Białystok, Poland), MMP-9 homozygous knockout mice (MMP-9 KO), and their WT siblings (MMP-9 WT) on a C57BL/6J background [47]. All mice were maintained in the Animal House of the Nencki Institute. Animals were housed in individual cages under controlled environment (temperature 22 ± 1°C, humidity 50-60%, with free access to food and water and a 12 h light/dark cycle). All procedures were performed in accordance with the Animal Protection Act in Poland, directive 2010/63/EU, and were approved by the 1st Local Ethics Committee (Permissions Numbers: 383/2012; 609/2014).

### 2.2. Induction of TBI with CCI

**Figure 1:**
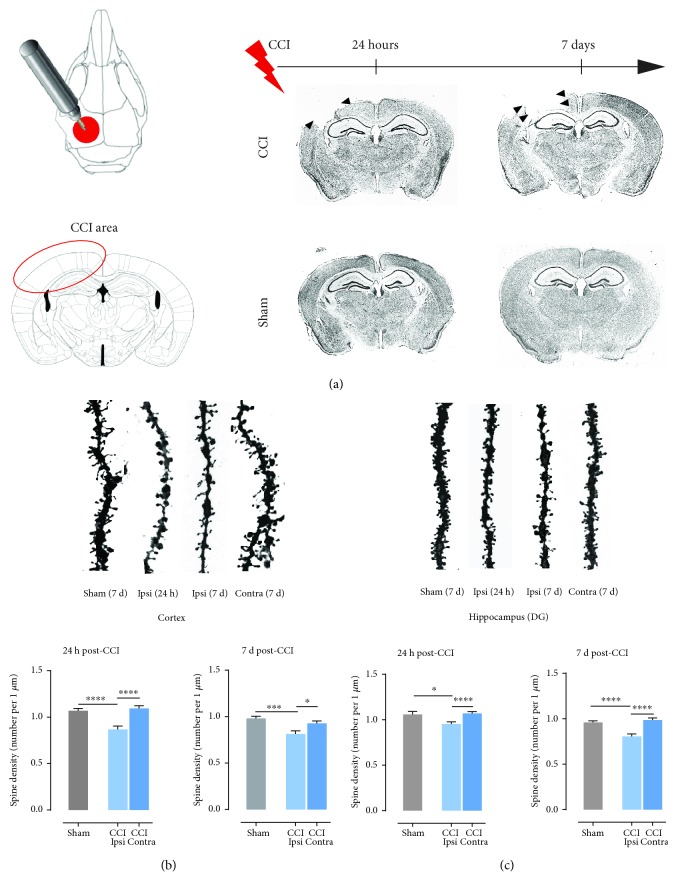
Decrease in spine density after controlled cortical impact (CCI). (a) Schematic representation of injured area. Nissl-stained brain sections from animals at 1 and 7 days after CCI (CCI: animals after controlled cortical impact; sham: animals subjected to craniectomy without cortical injury). (b) Spine density (number of spines per 1 *μ*m of dendrite length) in ipsi- and contralateral 2nd and 3rd cortex layers of C57Bl6/J mice, 1 and 7 days after CCI and sham procedures; right panel shows representative dendrites pictures. (c) Spine density in ipsi- and contralateral dentate gyrus of C57Bl6/J mice, 1 and 7 days after CCI and sham procedures; right panel shows representative dendrite pictures. Data are presented as mean ± SEM. Statistical analysis was carried out using one-way ANOVA followed by Tukey's post hoc test. Asterisks indicate statistical significance from the CCI and sham groups, respectively. ^∗^*P* < 0.05; ^∗∗∗^*P* ≤ 0.001; ^∗∗∗∗^*P* < 0.0001.

Mice were subjected to unilateral cortical contusion using the controlled cortical impact protocol [28, 46, 48] following anesthesia evoked with 4% isoflurane (Aerrane; Baxter, UK) in 100% oxygen with a delivery rate of 4 l/min. During the surgery, concentration of isoflurane was maintained at the level of 3% in 100% oxygen with delivery of 0.6 l/min (Combi Vet Anesthesia System; Rothacher; Switzerland). For deeper sedation, briefly before the injury, mice were injected subcutaneously with butorphanol (10 *μ*g/30 g body weight). After skull exposure by midline scalp incision, craniectomy was performed using a 5 mm ∅ trephine (Fine Science Tools FST; Germany) over the left parietotemporal cortex between the lambda and bregma (Figure 1(a)). The bone piece was carefully removed without disrupting the dura. For TBI execution, we used Leica Impact One device equipped with an electrically driven metallic piston controlled by a linear velocity displacement transducer (Leica Biosystems, KAWA.SKA; Poland). After craniectomy, the adjustable CCI equipment was mounted on the left stereotaxic arm at an angle of 20° from vertical. CCI was delivered according to the protocol [28] using the following parameters: ∅ 3 mm: flat tip; depth: 0.5 mm from the dura; velocity: 5 m/s, and dwell time: 100 ms. After injury, bleeding was strictly controlled, a piece of sterile plastic was placed over the craniectomy area, and the incision was sutured with nylon stitches (Sigmed; Poland). Next, the animals were returned to the heated home cages for postsurgical recovery. Sham-injured animals underwent identical anesthesia and craniectomy procedures, but were not subjected to CCI. The following number of animals was subjected to procedure per each time point: C57BL/6J (*n* = 5 CCI, *n* = 5 sham), MMP-9 WT (*n* = 5 CCI, *n* = 3 sham), and MMP-9 KO (*n* = 5 CCI, *n* = 3 sham).

### 2.3. Nissl Staining

To verify cerebral cortex degeneration, we performed Nissl staining. 24 hours and 7 days after the injury, mice were anesthetized and perfused with 0.37% sulfide solution (5 ml/min, 4°C) for 5 min followed by perfusion with 4% paraformaldehyde in 0.1 M sodium phosphate buffer, pH 7.4 (5 ml/min, 4°C) for 10 min. The brains were removed from the skull and postfixed in buffered 4% paraformaldehyde for 4 h at 4°C, and then cryoprotected in a solution containing 30% glycerol in 0.02 M potassium phosphate-buffered saline for 48 h. Samples were then frozen on dry ice and stored at -80°C. Frozen brains were sectioned in the coronal plane (40 *μ*m) with a sliding cryostat (Leica Biosystems, KAWA.SKA; Poland). The sections were mounted on microscope gelatin-covered slides, dried, and stained with cresyl violet. Pictures of Nissl-stained sections were taken using the light microscope Nikon Eclipse Ni equipped with PlanApo 2x objective.

### 2.4. Dendritic Spine Analysis

**Figure 2:**
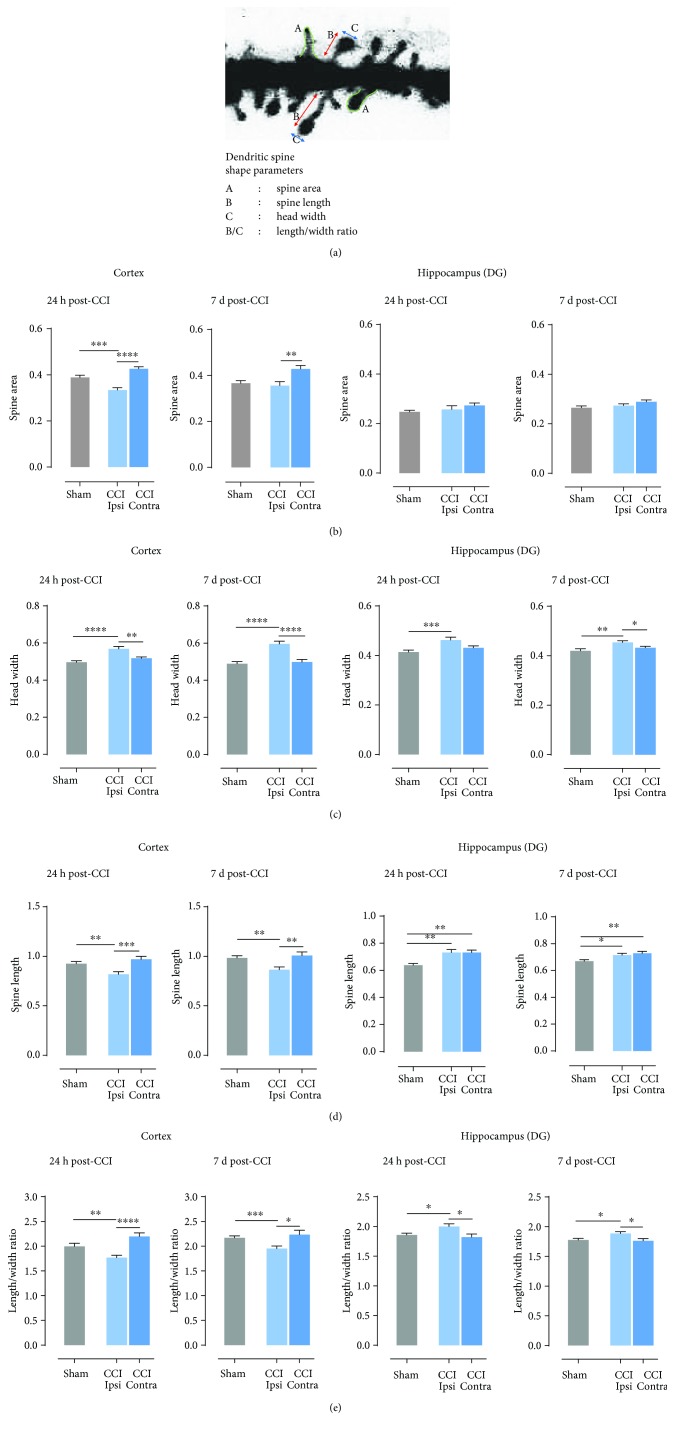
Time-dependent changes in dendritic spines shape after controlled cortical impact (CCI). (a) Spine shape parameters: A: spine area; B: spine length; C: head width; B/C: length/width ratio. (b) Spine area calculated in the ipsi- and contralateral cortex and hippocampus of C57Bl6/J mice, 1 and 7 days after CCI and sham procedures. (c) Head width calculated in the ipsi- and contralateral cortex and hippocampus of C57Bl6/J mice, 1 and 7 days after CCI and sham procedures. (d) Spine length calculated in the ipsi- and contralateral cortex and hippocampus of C57Bl6/J mice, 1 and 7 days after CCI and sham procedures. (e) Length/width ratio calculated in the ipsi- and contralateral cortex and hippocampus of C57Bl6/J mice, 1 and 7 days after CCI and sham-operated animals. Data are presented as mean ± SEM. Statistical analysis was carried out using one-way ANOVA followed by Tukey's post hoc test. Asterisks indicate statistical significance from the CCI and sham groups, respectively. ^∗^*P* < 0.05; ^∗∗^*P* ≤ 0.01; ^∗∗∗^*P* ≤ 0.001; ^∗∗∗∗^*P* < 0.00013.3.

Dendritic spines were visualized using lipophilic dye Dil (1,1′-dioctadecyl-3,3,3′,3′-tetramethylindocarbocyanine perchlorate, #D282 Life Technologies, Warsaw, Poland). 24 hours and 7 days after CCI, mice were sacrificed and their brains were collected. Next, they were cut into 130 *μ*m sections on vibratome (Leica VT 1000S, Leica Biosystems Nussloch GmbH, Wetzlar, Germany). Slices were processed for Dil staining. Random dendrite labeling was performed using 1.6 *μ*m tungsten particles (Bio-Rad, Hercules, CA, USA) coated with Dil. Dye was delivered to cells using Gene Gun (Bio-Rad). After staining, slices were fixed with 0.4% paraformaldehyde in phosphate-buffered saline (PBS; overnight at 4°C) and placed on microscopic slides. *Z*-stacks of dendrites from the 2nd and 3rd layers of the perilesional cortex and the dentate gyrus (DG) were acquired using the LSM780 confocal system equipped with 40x objective (Plan Apochromat 40x/1.4 Oil DIC) (Zeiss, Poznań, Poland). Dil emission was excited using a HeNe 594 nm laser. For each image, the following parameters were applied: 70 nm pixel size, 300 nm *Z*-intervals, averaging 4. Maximum intensity projections of *Z*-stacks covering the length of dendrite were analyzed using semiautomatic SpineMagick! software [49]. It allows marking dendritic spine head and base manually. Next, the software marks automatically spine edges that can be adjusted manually to fully reflect the spine shape [49]. For each animal, 5-7 single dendrites from selected brain areas (one dendrite per neuron per image) were analyzed. First, dendritic spine density was calculated. In the next step, dendritic spines were examined according to the following morphological parameters: spine area, head width, spine length, and a scale-free parameter - the length divided by the width (length to width ratio) (Figure 2(a)). This parameter reflects the spine shape—the higher the ratio, the more filopodial spine is.

To determine group size, we used assumption on the basis of sample size determination and the following equation:

(1) $$\begin{array}{c} n=1+2C{\left(\frac{s}{d}\right)}^{2}, \end{array}$$

where *n* is the group size, *C* is a constant dependent on the value of *α* and power selected (here it equals 10.51 for 0.9 power and 0.05 significance level), *s* is an estimate of the population standard deviation of the variable, and *d* is the magnitude of the difference. With the above-estimated values, the group size is 10. In our experiments, we increased the group size and it varied between 15 and 25 depending on the experiment (5 to 7 pictures per animal, 3-5 animals per group).

### 2.5. Statistical Analyses

All results are expressed as mean ± SEM. The appropriate tests were chosen (see below), taking into account whether data had normal distribution and equal variation. All analyses were conducted using GraphPad Prism, version 7.02 (GraphPad Software Inc., La Jolla, CA). Differences between the experimental groups were considered significant if the type 1 error was less than 5%.

## 3. Results

### 3.1. Decrease in Spine Density in the Cerebral Cortex and the Hippocampus Evoked by Controlled Cortical Impact (CCI)

To induce TBI in mice, we used controlled cortical impact (CCI) as described by Bolkvadze and Pitkänen [46]. To describe morphological changes evoked by injury, the brains were collected 24 hours and 7 days post-CCI. Control animals (sham-operated) were subjected to craniectomy only and sacrificed together with the animals that underwent CCI. The Nissl staining revealed time-dependent cerebral cortex degeneration within the injured area (Figure 1(a)). In sham-operated animals, hardly any tissue damage was observed. A separate batch of animals was used to perform dendritic spine analyses. Animals were subjected to CCI or sham surgery. Twenty-four hours or 7 days later, their brains were collected to perform analyses of dendritic spines from ipsi- and contralateral sides. For this, we used lipophilic dye that incorporates into cell membranes and marks the whole cell contour. Sections were imaged using a confocal microscope, as described in Materials and Methods. Spine density was calculated as a number of protrusions per 1 *μ*m of dendrite length. Twenty-four hours and 7 days after CCI, spine density was decreased in the 2nd and 3rd layers of the ipsilateral cerebral cortex, as compared to the contralateral hemisphere (24 h ^∗∗∗∗^*P* < 0.0001; 7 d ^∗^*P* = 0.0135) and sham animals (24 h ^∗∗∗∗^*P* < 0.0001; 7 d ^∗∗∗^*P* = 0.0003; Figure 2(b)). Similar effect was observed in the DG where 24 hours and 7 days after TBI spine density were significantly lower compared to the contralateral side (24 h ^∗^*P* < 0.0001; 7 d ^∗^*P* = 0.0001; Figure 1(c)) and sham-operated animals (24 h ^∗^*P* = 0.0251; 7 d ^∗∗∗∗^*P* < 0.0001; Figure 1(c)).

### 3.2. Dendritic Spines Become Shorter and Wider in Injured Cerebral Cortex Area

Next, we aimed at more detailed analysis of morphological alterations following trauma. For this, we used a semiautomatic software to evaluate dendritic spine shapes on the basis of the following parameters: length, head width, and length to width ratio that describes the spine shape, with its increase reflecting filopodial shape (Figure 2(a)). The parameters were measured at two time points, 1 and 7 days after CCI. Dendritic spines shrank in the ipsilateral cerebral cortex following trauma as their areas were smaller both 24 hours and 7 days after brain injury, compared to the contralateral cortex (24 h ^∗∗∗∗^*P* < 0.0001; 7 d ^∗∗^*P* = 0.0057; Figure 1(b)). Significant difference between ipsilateral hemisphere and sham-operated animals was observed only after 24 hours (^∗∗∗^*P* = 0.0005), while in the DG, no significant differences were observed (Figure 2(b)). Head width (width at the widest point of the spine) increased both in the cortex and DG in injured hemispheres, compared to the contralateral hemisphere (cortex: 24 h ^∗∗^*P* < 0.0015; 7 d ^∗∗∗∗^*P* > 0.0001; DG: 7 d ^∗^*P* = 0.033) and animals that underwent sham surgeries (cortex: 24 h ^∗∗∗∗^*P* > 0.0001; 7 d ^∗∗∗∗^*P* > 0.0001; DG: 24 h ^∗∗∗^*P* = 0.0005; 7 d ^∗∗^*P* = 0.0086) (Figure 2(c)). Next parameter, spine length, reflects the distance from the bottom to the top of the spine (Figure 2(d)). In the ipsilateral cortex, spine length was decreased compared to the contralateral hemisphere in both time points (24 h ^∗∗∗^*P* = 0.0006; 7 d ^∗∗^*P* = 0.0097) and sham-operated animals (24 h ^∗∗∗^*P* = 0.0021; 7 d ^∗^*P* = 0.0043). In contrast, in the ipsilateral DG, spines were longer than in animals after sham operation (24 h ^∗∗^*P* = 0.0021; 7 d ^∗^*P* = 0.0166), and no difference compared to the contralateral hemisphere was observed. Decrease of the ratio between the length and width of the spine was observed in the ipsilateral cerebral cortex when compared to the contralateral side (Figure 2(e), 24 h: ^∗∗^*P* < 0.0001; 7 d ^∗^*P* = 0.0182) and sham animals (24 h: ^∗∗^*P* = 0.0061; 7 d ^∗∗∗^*P* = 0.0010). While in the ipsilateral DG, opposite effect was noticed, where the length/width ratio increases in response to TBI compared to contralateral DG (24 h ^∗^*P* = 0.0166; 7 d ^∗^*P* = 0.026) as well as in comparison to sham animals (24 h ^∗^*P* = 0.0402; 7 d ^∗^*P* = 0.037; Figure 2(e)).

### 3.3. Deficiency of MMP-9 Impairs the Effect of Brain Injury on Spine Density Decline

**Figure 3:**
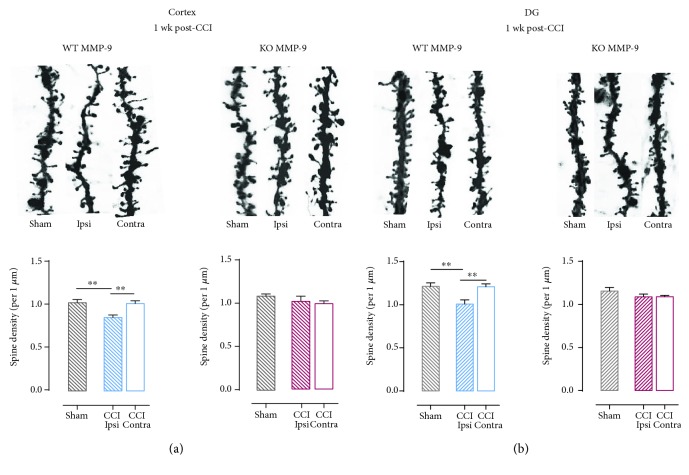
Effect of lack of functional MMP-9 on spine density in animals after controlled cortical impact (CCI). (a) Spine density in ipsi- and contralateral 2nd and 3rd cortex layers of animals with different *mmp-9* gene expression levels 1 week after CCI and sham surgeries; upper panel shows representative dendrites pictures. (b) Spine density in the ipsi- and contralateral dentate gyrus of animals with different *mmp-9* gene expression levels 1 week after CCI and sham surgeries; upper panel shows representative dendrite pictures. Data are presented as mean ± SEM. Statistical analysis was carried out using one-way ANOVA followed by Tukey's post hoc test. Asterisks indicate statistical significance from the CCI and sham groups, respectively. ^∗∗^*P* ≤ 0.01.

Since MMP-9 is one of the key modulators of dendritic spines shape and its role in TBI and subsequent epileptogenesis has been recently highlighted [28], we set out to evaluate whether the lack of MMP-9 affects morphological changes observed in WT animals. For this, we used mice missing MMP-9 (MMP-9 KO) and their wild-type littermates (WT MMP-9). We focused on 7-day post-CCI time point. As we reported in the previous report, MMP-9 activity was the highest during first week after brain injury [28]. After CCI in WT and KO MMP-9 animals, we analyzed dendritic spines as described above. 1 week after the brain injury, spine density was decreased in the ipsilateral cerebral cortex of WT animals as compared to the contralateral side of the injured brain (^∗∗^*P* = 0.0011; Figure 3(a)) and sham-operated animals (^∗∗^*P* = 0.0067; Figure 3(a)). However, this phenomenon was not observed in MMP-9 KO mice (Figures 3(a) and 3(b)).

### 3.4. MMP-9 Is Required for Spine Shape Remodeling upon Injury

**Figure 4:**
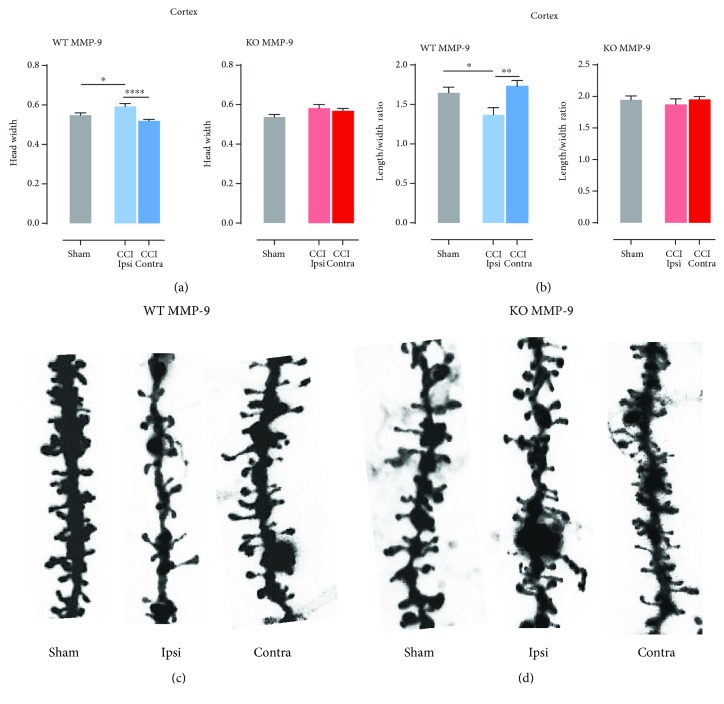
Effects of MMP-9 on spine shape changes in the cerebral cortex layers of animals 1 week post-CCI. (a) Head width calculated in the ipsi- and contralateral cortex and hippocampus of C57Bl6/J mice, 7 days after CCI and sham procedures. (b) Length/width ratio calculated in the ipsi- and contralateral cortex and hippocampus of C57Bl6/J mice, 7 days after CCI and sham-operated animals. (c) Dendrite pictures from the ipsi- and contralateral cortex and sham animals. (d) Representative dendrite pictures from the ipsi- and contralateral cortex and sham animals. Data are presented as mean ± SEM. Statistical analysis was carried out using one-way ANOVA followed by Tukey's post hoc test. Asterisks indicate statistical significance from the CCI and sham groups, respectively. ^∗^*P* < 0.05; ^∗∗^*P* ≤ 0.01.

**Figure 5:**
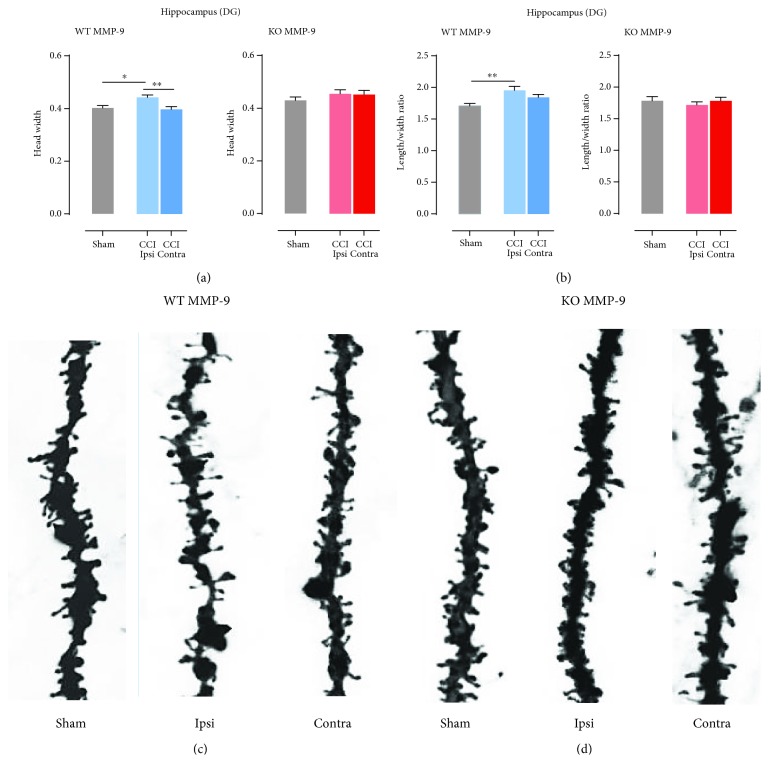
Effects of MMP-9 on spine shape changes in the dentate gyrus of animals 1 week post-CCI. (a) Head width calculated in the ipsi- and contralateral hippocampus of C57Bl6/J mice, 7 days after CCI and sham procedures. (b) Length/width ratio calculated in the ipsi- and contralateral hippocampus of C57Bl6/J mice, 7 days after CCI and sham-operated animals. (c) Dendrite pictures from the ipsi- and contralateral dentate gyrus and sham animals. (d) Representative dendrite pictures from the ipsi- and contralateral dentate gyrus and sham animals. Data are presented as mean ± SEM. Statistical analysis was carried out using one-way ANOVA followed by Tukey's post hoc test. Asterisk indicate statistical significance from the CCI and sham groups, respectively. ^∗^*P* < 0.05; ^∗∗^*P* ≤ 0.01.

To further unravel the influence of MMP-9 on the postinjury morphological changes of dendritic spines, we analyzed basic spine parameters (Figure 2(a)). We focused on length to width ratio and spine head width as these parameters indicate whether protrusions undergo plastic changes. In WT animals, in the ipsilateral cerebral cortex area, we observed increase in head width compared to contralateral hemisphere (^∗∗∗∗^*P* < 0.0001; Figure 4(a)) and sham-operated animals (^∗^*P* = 0.0477; Figure 4(a)). Similarly, to the cerebral cortex, in the ipsilateral dentate gyrus of hippocampus WT MMP-9 mice, head width was significantly bigger compared to the contralateral hippocampus (^∗∗^*P* = 0.0020; Figure 5(a)) and sham-operated animals (^∗∗^*P* = 0.0061; Figure 5(a)). On the contrary, in MMP-9 KO animals, following CCI, no alterations in dendritic spine morphology were detected in both analyzed structures (Figures 4(a) and 5(a)). In the injured cerebral cortex, WT MMP-9 mice length/width ratio decreased, while in the ipsilateral dentate gyrus increased (Figures 4(b) and 5(b)). The changes in the ipsilateral cortex was significant compared to the contralateral side of the brain (^∗∗^*P* = 0.0048; Figure 4(b)) and sham animals (^∗^*P* = 0.0316; Figure 4(b)), whereas in MMP-9 KO mice, no differences were observed (Figure 4(b)). In the DG, ratio between the spine length and width in ipsilateral hemisphere was significantly higher compared only to sham animals (^∗∗^*P* = 0.0016; Figure 5(b)). Similarly, to the cortex, the changes between the CCI and sham groups were not significant (Figure 5(b)).

## 4. Discussion

Here, we show that traumatic brain injury caused by the controlled cortical impact induces acute (within 1 day post-TBI) changes in the dendritic spine number and morphology, as well as prolonged (up to 7 days after the injury) spine remodeling. Spine density decreases following brain trauma in the ipsilateral side both in the cerebral cortex and the dentate gyrus of the hippocampus. Following trauma dendritic spines in the ipsilateral cerebral cortex shrink, get shorter and their heads get wider, thus being converted into more mushroom-like shape. On the other hand, spines in the DG on the ipsilateral side get longer and thinner, assuming more filopodial form. Lack of MMP-9 activity in the brain abrogates the effects evoked by the trauma, both as far as the spine dynamics (reflected by changes in the density) and morphological plasticity are concerned.

Dendritic spines are protrusions containing the majority of excitatory synapses, thus gating inputs received by the nerve cell [4, 50]. The density and morphology of dendritic spines are regulated by synaptic activity, and so spines undergo dynamic turnover throughout life. Filopodia-shaped spines are more prominent in the developing brain and are considered “immature” [51]. Some reports indicate that these are spine precursors during synapse formation [52]. Spines considered “mature” are more mushroom-shaped, allowing stabilizing the spine by gathering more neurotransmitter receptors in the head [53]. Other feature of such shape is that the narrow spine neck might also compartmentalize calcium necessary for synaptic transmission to occur [54]. When the brain is challenged by injury, spines respond accordingly to maintain milieu homeostasis [7].

First, we measured dendritic spine density. Spine number has been shown to be related to overall synaptic activity, e.g., in the hippocampus transient enhancement of dendritic spine density accompanies early long-term potentiation (LTP) in the dentate gyrus [55]. Previous studies on dendritic spine plasticity evoked by TBI in animal models showed decreased spine density in the hippocampus and the cerebral cortex at various times after brain injury (24 hours to 35 days) [9, 11, 13, 16]. Furthermore, one study expands these observations up to 1.5 years, showing changes that last not only shortly after brain injury but also continue into chronic stages of TBI. Moreover, they take place in widespread regions beyond the site of acute trauma [12].

Dendritic spine loss is accompanied by dendrite deformation and swelling [15]. Our results are essentially in agreement with the abovementioned data; however, they extend those observations by describing the detailed shape parameters. In the present study, we show that 24-hour postinjury dendritic spine density decreases in the ipsilateral side in the cortex and the hippocampus, which is consistent with the previous report [8, 11]. We further demonstrate that 7 days after injury the number of spines per dendrite length is still decreased in the cortex and the DG. Dendritic spine loss one-week posttrauma was also shown in the CA1 of the hippocampus [12]. Since our TBI conditions were far less severe, we conclude that even mild injury then can result in substantial changes in the brain.

Furthermore, we also show a detailed morphological analysis of dendritic spines in the cortex and hippocampus after traumatic brain injury. Here, we demonstrate that after brain trauma induced by CCI, dendritic spines in the cerebral cortex, ipsilaterally to the injury side, are getting shorter and their heads become wider, when compared to sham-operated animals and the contralateral side of the injured cerebral cortex. Overall, the spine plasticity observed in the cerebral cortex ipsilateral to the injury and in a deeper located DG showed similar pattern of spine morphological alterations, except for the length/width ratio. This morphological parameter is believed to reflect spine maturity [4, 39, 52]. The greater this value, the spine is more filopodial-shaped, i.e., immature. This finding deserves a special comment. The filopodia-like spines are presumably prone to support initiation of synaptic plasticity processes [4, 36, 50]. Therefore, this result may suggest that DG may undergo synaptic plasticity that may predispose this brain structure to support epileptogenesis that is a frequent consequence of TBI [56, 57].

In the present study, we show that TBI-driven dynamics and morphological plasticity of dendritic spines are MMP-9-dependent. Increases in MMP-9 following TBI have previously been reported both in the animal brain and human cerebrospinal fluid, blood plasma, and serum [9, 30, 44–47, 58, 59]. The possible role of MMP-9 in controlling spine dynamics has been previously demonstrated in the DG, following treatment with excitotoxic kainic acid, where MMP-9 KO mice were found to be resistant to spine loss [60, 61]. Similarly, recently, Nagaoka et al. [62] have reported that MMP-9 controls spine dynamics in the neocortex of fragile X mental retardation protein KO mice.

Furthermore, in our study, missing MMP-9 has entirely abrogated dendritic spine morphological plasticity provoked by TBI, both in the cerebral cortex ipsilateral to the injury and in the dentate gyrus. This finding goes well along with multiple data showing a pivotal role of MMP-9 in regulation of dendritic spine size and shape [29, 63, 64].

Finally, we shall stress that previous studies [28, 40] demonstrated that MMP-9 KO mice brain showed limited brain damage after CCI. However, the extend of this protection against brain damage (MMP-9 KO still demonstrated almost 40% of the cortical injury area as compared to WT) does not seem to explain the entire abrogation of TBI effects on the spines in the MMP-9 KO brains.

## 5. Conclusions

Herein, we have provided a detailed analysis of TBI-evoked density and morphological plasticity of the dendritic spines. We have found that in result of the injury, there is a decrease in spine density both very close (ipsilateral cerebral cortex) and more distal (hippocampal dentate gyrus on the ipsilateral side) to the locus of the injury. However, the spines located on neurons close to the injury assume more mushroom-like shape, whereas those in DG become more filopodia-like. Missing MMP-9 previously shown to exert control of the spine density and morphology abrogated the aforementioned plasticity entirely. Considering the previously reported role of MMP-9 in posttraumatic epileptogenesis (PTE) that might be supported by abnormal synaptic plasticity, and a well-documented role of this enzyme in the plasticity of dendritic spines, it is tempting to suggest that MMP-9-dependent dendritic spine dynamics and morphological plasticity contribute to PTE.

## Data Availability

Previously reported (MMP-9 activity after CCI; role of MMP-9 in posttraumatic epileptogenesis) data were used to support this study and are available at doi:10.1007/s12035-018-1061-5. These prior studies (and datasets) are cited at relevant places within the text as reference [28].
